# What drove changes in alcohol sales during the COVID‐19 pandemic in Czechia? An interrupted time series analyses

**DOI:** 10.1111/dar.14035

**Published:** 2025-03-03

**Authors:** Benjamin Petruželka, Miroslav Barták, Vladimir Rogalewicz, Thomas F. Babor

**Affiliations:** ^1^ Department of Addictology First Faculty of Medicine, Charles University Prague Czech Republic; ^2^ Department of Addictology General University Hospital in Prague Prague Czech Republic; ^3^ Department of Biomedical Technology Faculty of Biomedical Engineering, Czech Technical University in Prague Kladno Czech Republic; ^4^ Department of Public Health Sciences University of Connecticut School of Medicine Farmington USA

**Keywords:** alcohol availability, alcohol sales, COVID‐19, time series, tourism

## Abstract

**Introduction:**

Research on the effects of the COVID‐19 pandemic on alcohol sales and per capita consumption show mixed findings. The present study of alcohol sales in Czechia attempts to account for this heterogeneity by investigating three types of interventions: (i) limits on the movement of people into the country (i.e., tourism); (ii) social mobility and assembly restrictions; and (iii) restrictions on alcohol sales.

**Methods:**

We used general additive models to assess the relationship between alcohol sales and COVID‐19‐related restrictions that were specific to alcohol outlets and general restrictions measured by the Government Response Stringency Index. New COVID‐19 cases and the number of overnight stays by foreign tourists were also included in the models.

**Results:**

The analysis of total sales revenues show that the overall amount of alcohol sold in the Czech Republic decreased due to the COVID‐19 related measures and the decrease is best explained by the Government Response Stringency Index. We did not find any relationship between alcohol sales and the number of new COVID‐19 cases.

**Discussion and Conclusions:**

The decline in total alcohol sales resulting from the COVID‐19 restrictions was not just the result of reduced tourism and restrictions on physical availability of alcohol, but also of restrictions limiting social encounters. This should be considered in future research comparing the trends in different countries and incorporated into plans for controlling the spread of communicable diseases in future epidemics.

**Key Points**

- We used general additive models to assess the relation between alcohol sales and COVID‐19‐related restrictions (specific to alcohol outlets and general restrictions represented by the Government Response Stringency Index), as well as new COVID‐19 cases and number of overnight stays by foreign tourists were also included in the models.
- The analysis of total sales revenues shows that the overall amount of alcohol sold in the Czech Republic decreased with the imposition of COVID‐19‐related measures and the decrease is best explained by Government Response Stringency Index.
- The decline in total alcohol sales resulting from the COVID‐19 restrictions was not just the result of reduced tourism and restrictions on physical availability of alcohol, but also of restrictions limiting social encounters.

## 1 INTRODUCTION

Reviews and meta‐analyses focused on studies of the influence of the COVID‐19 pandemic on alcohol use show heterogeneous results [1, 2, 3]. The results differ according to patterns of alcohol consumption in the countries under investigation [2, 3]. In some countries heavy episodic drinking and the proportion of people with problematic alcohol use may have increased, even while overall alcohol use may have decreased [3]. In Europe more people reduced their alcohol use than increased. These changes in alcohol use may be associated with pre‐pandemic drinking levels, with risky drinkers increasing the level of drinking [2]. However, the influence of public health measures on consumption is not clear [2]. Alcohol sales data allow us to examine the influence of national measures because they are usually collected continuously and are not limited to only one or a few time points, as surveys usually are. Thus, an analysis of changes in alcohol sales during the COVID‐19 pandemic can complement prior research based on survey studies of self‐reported alcohol consumption during the COVID‐19 pandemic [4, 5, 6, 7, 8] or studies of specific mortality rates that were associated with alcohol diagnoses during and after the COVID pandemic [9, 10, 11, 12].

The COVID‐19 pandemic was associated with a number of changes in people's purchasing behaviour including alcohol sales [13]. Similarly, as in the case of surveys, the changes in alcohol sales show mixed results across countries with some studies indicating an increase in alcohol sales [5, 6, 14, 15, 16, 17, 18], others indicating a decrease in alcohol sales [19], and still others indicating no change [20]. It is therefore important to focus on the factors influencing alcohol sales to explain the differential impact of the COVID‐19 pandemic on alcohol consumption and alcohol‐related problems at the national level.

In the United States, one [6] study showed an increase in alcohol sales in the early phase of the pandemic. Similar results were obtained by another study [5]. A Canadian study [14] identified substantial increases in alcohol sales during the COVID‐19 lockdowns compared to the period before the COVID‐19 pandemic. Conversely, another Canadian study [19] found that alcohol‐specific restrictions implemented in response to the COVID‐19 pandemic were associated with reduced consumption. However, the magnitude and direction of the changes were moderated by the level of deprivation in the region measured by the Canadian Index of Multiple Deprivation, which includes residential instability, ethno‐cultural composition, economic dependency and situational vulnerability—albeit inconsistently across various deprivation measures. Predictors of change in alcohol consumption that were highlighted in one review [1] included the drinking environment and social context (e.g., solitude, number of friends) as well as situational living factors (e.g., living single, number of children, type of dwelling, income and job changes) and individual difference factors such as depression and other mental health conditions. The decrease of drinking was also attributed to the effect of fewer social occasions [21].

A study of Finland, Norway and Sweden found no relevant change in alcohol sales during the pandemic years, while the recorded changes could only be attributed to seasonal changes in sales [20]. A British study from the first wave of the pandemic showed that the most deprived households purchased 29% more alcohol off‐trade, and that this increase was greater at the time of full pub closures than for the rest of the period analysed [16]. Leifman [15] analysed alcohol sales in the context of closed borders and COVID‐19 restrictions in several European countries. While they generally found an increase in sales, countries with cross‐border inflows recorded a decrease in the total amount of alcohol consumed per capita, because not all cross‐border purchases were replaced by domestic sales.

Another factor that may be related to alcohol sales and consumption during the pandemic is tourism. Alcohol consumption by tourists can be evaluated from several perspectives. One is the negative phenomena (e.g., public intoxication) that are mainly related to overtourism [22] Another is the effort by the hospitality sector to increase the number of tourists who come to a country to engage in “alcohol tourism” especially during sporting and entertainment events [23]. The impact of public health measures on alcohol consumption in the late‐night economy was mentioned several times in relationship to changes in criminality during the pandemic [24, 25]. The COVID‐19 pandemic and its related restrictions on tourism allow us to assess the influence of consumption by foreign tourists in Czechia. The most recent data available from 2019 on tourist alcohol per capita consumption for Czechia are estimated by the World Health Organization (WHO) as −0.1 [−0.2 to 0.1] [26]. WHO uses 3‐year averages in this data. According to WHO, tourist alcohol per capita consumption are the litres of pure alcohol which are consumed by tourists visiting a country in a calendar year [27]. The above‐mentioned figure is adjusted for the alcohol purchased and consumed when people are visiting countries other than their home country. The negative figure for Czechia means that total alcohol consumption of outbound tourists is lower than total alcohol consumption by inbound tourists, that is, Czech tourists drink less alcohol in foreign countries than foreign tourists in Czechia. The indicator shows that the total consumption in Czechia was slightly increased by foreign tourist alcohol consumption in the year before pandemic.

The present study of alcohol sales in Czechia attempts to improve our limited knowledge of factors that influence alcohol consumption during the COVID 19 pandemic by investigating the contributions of three types of broad behavioural interventions: (i) limits on the movement of people into the country for personal or business/professional purposes (i.e., tourism), which often involves alcohol consumption; (ii) social mobility and assembly restrictions, which could limit opportunities for hospitality and social drinking; and (iii) restrictions on alcohol sales at on‐premise and/or off‐premise alcohol outlets. According to the alcohol availability theory [28], each of these public health measures should reduce the overall amount of alcohol sold and consumed at a national level in Czechia during the COVID 19 pandemic. To the best of our knowledge, our study is unique in combining different data sources (see the Methods section for details) in a time series analysis that provides a deeper understanding of factors influencing alcohol sales. And no other analysis has used time series to describe the impact of COVID‐19‐related regulations by means of the Government Response Stringency Index (GRSI) [29], while taking into account changes in the number of overnight tourists. Furthermore, we used number of new COVID‐19 cases and employed two independent sources of information about alcohol sales and two indicators of COVID‐19 measures, one focusing on the alcohol outlets and other on limiting movement and social gatherings.

Similar to other European countries, alcohol was consistently available in the Czech Republic during the COVID‐19 pandemic in supermarkets, grocery stores, gas stations and e‐shop services, but unprecedented governmental measures limiting social interactions between individuals and disrupting daily life in the country likely affected alcohol sales. The main issues believed to have been reflected in the pattern of alcohol sales in Czechia are closure of ‘unnecessary’ services including restaurants, bars and cafes, bans on drinking alcohol in public places, restrictions on gatherings and the movement of residents, allowances for working from home, as well as complete curfews, closed borders, restrictions on cross‐border movement of tourists and major global restrictions on tourism implemented during various periods of the pandemic. It should be noted that these measures were enforced by the police and other state authorities. Table A1 summarises the main limitations on life in Czechia related to the above‐mentioned areas (for details see [30]).

## 2 METHODS

We used general additive models to assess the relation between alcohol sales and COVID‐19‐related variables and number of overnight foreign tourists.

### 2.1 Data sources

#### 2.1.1 Alcohol sales

To capture alcohol sales, we used two data sources: Ministry of Finance of the Czech Republic, which provided total sales revenues, and Nielsen IQ, which provided retail sales data. Both data sets have their advantages and disadvantages. The Ministry of Finance records data as the tax is collected, that is, when alcohol is stored (not when alcohol is sold) and provides full coverage of the legal (i.e. recorded) domestic alcohol sales. The Nielsen IQ data set covers sales in all kinds of shops except petrol stations and e‐commerce and records data when the alcohol is sold. In this regard, we can expect Nielsen IQ to be more accurate regarding the time of actual consumption of alcohol. Other differences between the two data sets are the coverage of the alcohol sales points and specific kinds of alcohol included. The Ministry of Finance reports data in litres of liquid in the following categories: ethanol, fruit distillates, beer, still wine, sparkling wine and intermediate products. The Nielsen IQ data set covers sales in litres of liquid in all kinds of shops except petrol stations and e‐commerce. Thus, sales in the hospitality sector are not covered. Information about categories of alcohol was not monitored in shops. Beer and distillates were covered in hypermarkets, supermarkets and in smaller shops. Sparkling and still wines were covered only in hypermarkets and supermarkets. The data from Nielsen IQ were only available from 2019 to 2022, while data we use from the Ministry of Finance start in 2013 and end in 2022. The two data sets are compared in Table 1.

**TABLE 1:** Comparison of data sets used to measure alcohol sales.

Source of data	Ministry of Finance of the Czech Republic	Nielsen IQ
	Total sales revenues	Retail sales
Place of recording	As the tax is collected, that is when alcohol is stored	Sales in retail shops except petrol stations and e‐commerce; the sales in the hospitality sector are not covered
Alcohol included	All alcohol in the following categories: ethanol, fruit distillates, beer, still wine, sparkling wine, and intermediate products	Beer and distillates—in hypermarkets, supermarkets and smaller shops. Sparkling and still wines—only in hypermarkets and supermarkets
Units	Litres of liquid or of ethanol	Litres of liquid
Time period available	2013–2022	2019–2022
Aggregation	Monthly	Monthly

#### 2.1.2 Data sources for COVID‐19‐related variables and tourism

We used two sources of COVID‐related information: (i) publicly available data on the number of COVID cases per day and the GRSI, these were downloaded from https://ourworldindata.org/covid-cases [31]; and (ii) description of restrictions on alcohol sales by Rogalewicz et al. [30]. The GRSI is a composite measure based on school closures, workplace closures, international movement restrictions, cancellation of public events and gatherings, public transport closures, and stay‐at‐home requirements [29]. The GRSI is focused on the government responses to COVID‐19, mostly the responses that limited the movement of individuals and social gatherings. Thus, it is a good indicator of these restrictions rather than those influencing alcohol outlets.

The data about influence of tourism was taken from the Czech Statistical Office and their public database [32].

### 2.2 Variables

#### 2.2.1 Outcomes

**FIGURE A2:**
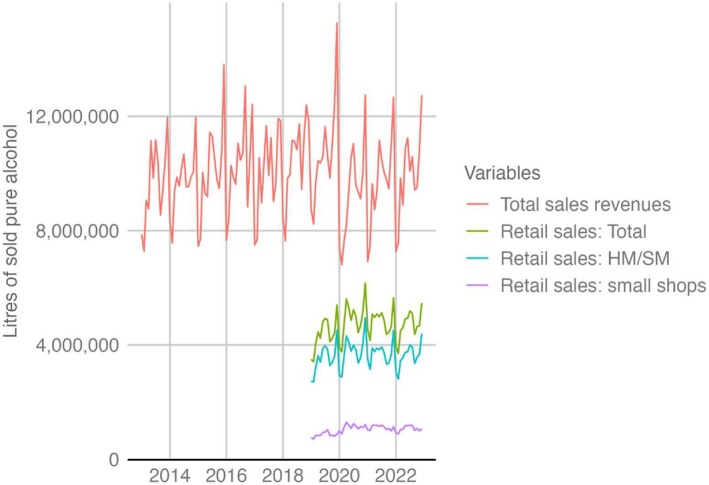
Alcohol sales in Czech Republic.

Regarding the measurement of alcohol sales, we constructed new variables in two steps. First, because the data were provided in litres of liquid, we had to perform recalculations to obtain volumes of pure alcohol. For the conversion, we used the following standard estimates of the pure alcohol content in 1 L of liquid: beer 4.5%, spirits 38%, still wine 12%, sparkling wine 12%. In the case of alcohol sales revenues provided by Ministry of Finance, the data about ethanol and fruit distillates were provided in litres of pure alcohol thus requiring no recalculation. The second step was to construct totals. In the case of data from the Ministry of Finance, we constructed totals without any problem. However, in the alcohol retail sales data provided by Nielsen, as noted above, some kinds of shops and alcohol types are not covered, and thus we decided to construct totals for each segment of shops and include only beer and distillates monitored across all segments (total retail sales, hypermarkets and supermarkets, small shops). See the appendices for a chart of these time series (Figure A2).

#### 2.2.2 Intervention variables

**FIGURE A3:**
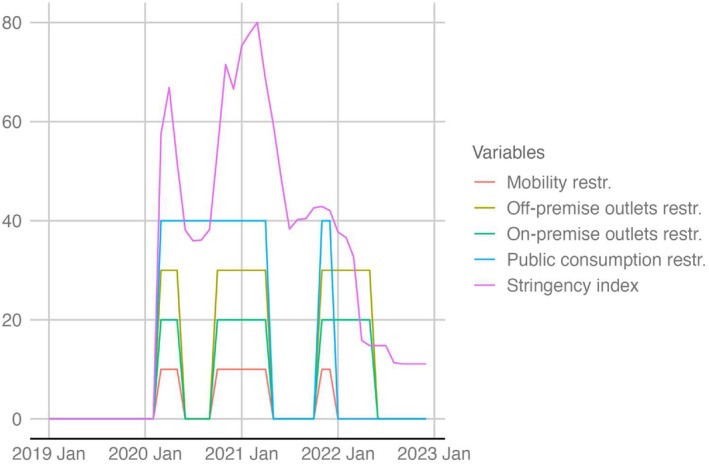
COVID‐19 related restrictions.

**FIGURE A4:**
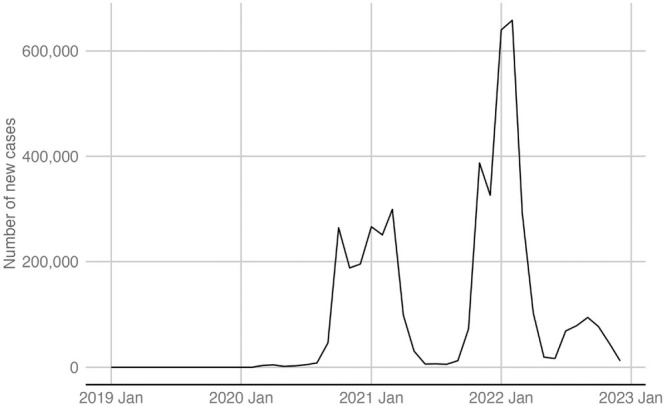
Number of new COVID‐19 cases.

We constructed new intervention variables based on the above‐described information about COVID‐19‐related interventions in Czechia. The restrictions on mobility and assembly were largely applied in a similar period, when the values of the GRSI index increased, and therefore we did not use it further. The restrictions influencing on‐premise alcohol outlets and off‐premise alcohol outlets were applied in overlapping periods of time, and therefore we created only one intervention variable capturing the restrictions on alcohol outlets. The slope effect was not considered because interventions were in effect for shorter periods of time and including the slope effect would be difficult. We also used number of COVID‐19 cases as an intervention variable. Furthermore, we constructed new variables with a 1‐month lag. The lag was based on the study by Stockwell et al. [18] describing the same situation in Canada. They showed that a lag exists (primarily identified as 2 weeks) between the number of COVID‐19 cases and alcohol sales data. In our study, the most appropriate time interval was considered to be 1 month. Shorter lags are impossible in the model due to the use of monthly aggregated data. In the case of policy interventions, the recommendation is to consider use of a lag [33]. We assume that the effect of the policy might be delayed because consumers were cautious at the beginning of the interventions in Czechia. Although consumers might have stocked up with alcohol at the beginning of the period, it is not reasonable to assume that this would affect the sales for longer time than 1 month. See the appendices for a chart of these time series (Figures A3 and A4).

**FIGURE A5:**
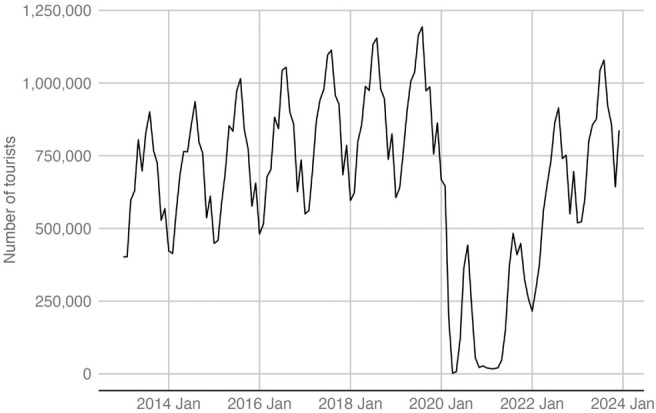
Number of foreign overnight tourists.

The tourism data were not modified because we do not assume that there would be any meaningful lag and the data were provided in monthly aggregation. See the appendices for a chart of these time series (Figure A5).

#### 2.2.3 Other variables

We also included other variables in the models, which were a trend variable (capturing the overall order of the series) and a seasonal variable (capturing the cyclic order of the series—months).

### 2.3 Statistical analyses

The analysis and management of data were carried out using R [34]. We utilised the mgcv package for the time series analysis. All time series that we examined and the related R codes are openly available in Data S1, Supporting Information.

We performed the time series models with function gamm(), which allows use of autocorrelation, specified by p and q terms. We did not use random effects and therefore we used the Generalised Additive Model (GAM) with the autoregressive moving‐average autocorrelation structure. Compared to conventional autoregressive integrated moving average models, GAM and Generalised Additive Mixed Model (GAMM) offer enhanced adaptability in representing seasonality and accounting for external influences. Because of these features, GAM and GAMM have become a popular choice for assessing the consequences of events such as the COVID‐19 outbreak [11, 35] and regulations related to alcohol consumption [36]. For all models, we used the Restricted Maximum Likelihood estimation method and the Gaussian distribution.

We used the same modelling strategy in the four alcohol sales variables: total sales revenues, total retail sales, hypermarket/supermarket retail sales and small shops retail sales. For each alcohol sales variable we fitted a baseline model, then we constructed a series of models which differed only in the intervention variables. All baseline models included a trend and a seasonal variable, as well as the specification of autocorrelation (if it was identified). The autocorrelation structure was specified using auto.arima() from the Forecast package [36]. Box.test() was used to test if residuals of the model did not exhibit autocorrelation. Furthermore, we visually checked this by using the autocorrelation and partial autocorrelation functions, namely acf() and pacf(). At each step of the modelling, we verified model properties by gam.check() anf k.check() (k‐check is a diagnostic test of whether the basis dimension for smooth is adequate). Furthermore, we visually inspected the splines. Based on these checks, we modified the models. We changed the smooth basis type, k value or decided if it is reasonable to use smooth. The final and optimal models and selection strategy are described in Appendix (A14).

After the baseline models were created, we constructed a series of models for each alcohol sales variable, which differed only in the intervention variables. Each model included only one intervention variables and their lags (in GRSI and COVID‐19 cases we also tested smooth and linear variants of predictors). Finally, for each alcohol sales variable, these models were compared using the akaike information criterion (AIC) criterion from the mgcv package [37, 38] to find the best model (see appendix for the syntax). Some of the intervention variables were found to be correlated (see Table A6), so we did not include them in a single model. Furthermore, we wanted to keep the same modelling strategy for the whole study.

## 3 RESULTS

Table 2 shows the volume of pure alcohol in litres sold between 2019 and 2022, including the percentage rate of change in sold volume of alcohol. There was an annual change in the case of the total sales revenues (data provided by Ministry of Finance), which shows first a sharp decrease of 13% between 2019 and 2020 and then a stabilisation of the trend. The 13% is equal to 16,956,811 L of pure alcohol, which is 1.97 L per inhabitant older than 18 years. Retail sales data show an increase between 2019 and 2020 and a stabilisation after that time with some decreases. The total retail sales (data provided by Nielsen IQ) increased by 12% (6,294,027 L of pure alcohol, which is 0.73 L per inhabitant older than 18 years) between 2019 and 2020, the highest increase was in small shops (31%, which is 3,181,863 L of pure alcohol and 0.37 L per inhabitant older than 18 years).

**TABLE 2:** Volume of sold pure alcohol in litres between 2019 and 2022.

	2019	2020	2021	2022	Change between 2019 and 2020 (%)	Change between 2020 and 2021 (%)	Change between 2021 and 2022 (%)
Total sales revenues	128,889,885	111,933,074	116,726,414	118,837,809	−13.156045	4.282327	1.808841
Retail sales: total	52,450,233	58,890,572	57,898,383	56,068,290	12.278951	−1.684800	−3.160872
Retail sales: HM/SM	42,186,158	45,376,245	44,549,589	43,289,656	7.561928	−1.821781	−2.828160
Retail sales: small shops	10,264,075	13,514,326	13,348,794	12,778,634	31.666289	−1.224866	−4.271248

Abbreviations: HM, hypermarkets; SM, supermarkets.

Table 3 shows the results of modelling. It does not include information about the models which were not used any further because the smooth did not provide suitable model. The optimal models and modelling decisions are described in more detail in Appendix (A14).

**TABLE 3:** Results of modelling.

Models		*p*‐value^a^	Parameter estimate/rate of change^b^	CI (95%): lower^c^	CI (95%): upper^c^	AIC
Total sales revenues	GRSI: linear	<0.001	−17,697	−25,461	−9933	3535.759
	GRSI: linear and lag	0.001	−13,706	−22,049	−5362	3543.421
	GRSI: smoothed	<0.001	−13,141	–	–	3530.008
	GRSI: smoothed and lag	0.008	−12,178	–	–	3538.726
	Outlet restrictions: linear	0.010	−566,105	−1,055,600	−76,610	3535.238
	Outlet restrictions: linear and lag	0.112	–	–	–	–
	COVID‐19 cases: linear	0.342	–	–	–	–
	COVID‐19 cases: linear and lag	0.306	–	–	–	–
	COVID‐19 cases: smoothed	–	–	–	–	–
	COVID‐19 cases: smoothed and lag	–	–	–	–	–
	Number of foreign tourists: linear	<0.001	1.323	0.900	1.756	3551.619
	Number of foreign tourists: smoothed	–	–	–	–	–
Retail sales: total	GRSI: linear	<0.001	11,136	6913	15,358	1251.686
	GRSI: linear and lag	0.001	8390	3530	13,249	1261.252
	GRSI: smoothed	<0.001	11,211	–	–	1243.613
	GRSI: smoothed and lag	–	–	–	–	–
	Outlet restrictions: linear	0.006	291,841	91,962	491,718	1256.720
	Outlet restrictions: linear and lag	0.023	245,064	37,715	452,412	1258.322
	COVID‐19 cases: linear	0.481	–	–	–	–
	COVID‐19 cases: linear and lag	0.766	–	–	–	–
	COVID‐19 cases: smoothed	–	–	–	–	–
	COVID‐19 cases: smoothed and lag	–	–	–	–	–
	Number of foreign tourists: linear	0.006	−0.573	−0.973	−0.172	1283.989
	Number of foreign tourists: smoothed	<0.001	−0.462	–	–	1252.084
Retail sales: HM/SM	GRSI: linear	<0.001	6066	3283	8850	1235.917
	GRSI: linear and lag	<0.001	5668	2240	9096	1239.555
	GRSI: smoothed	0.002	7285	–	–	1226.787
	GRSI: smoothed and lag	<0.001	4055	–	–	1232.322
	Outlet restrictions: linear	0.031	188,742	19,267	358,218	1237.242
	Outlet restrictions: linear and lag	0.020	202,278	34,837	369,720	1236.016
	COVID‐19 cases: linear	0.284	–	–	–	–
	COVID‐19 cases: linear and lag	0.819	–	–	–	–
	COVID‐19 cases: smoothed	0.360	–	–	–	–
	COVID‐19 cases: smoothed and lag	–	–	–	–	–
	Number of foreign tourists linear	0.001	−0.323	−0.601	−0.046	1263.531
	Number of foreign tourists: smoothed	<0.001	−0.301	–	–	1228.097
Retail sales: small shops	GRSI: linear	<0.001	2881	1330	4432	1146.535
	GRSI: linear and lag	0.090	–	–	–	–
	GRSI: smoothed	–	–	–	–	–
	GRSI: smoothed and lag	–	–	–	–	–
	Outlet restrictions: linear	0.042	67,674	2966	132,381	1146.623
	Outlet restrictions: linear and lag	0.533	–	–	–	–
	COVID‐19 cases: linear	0.197	–	–	–	–
	COVID‐19 cases: linear and lag	0.242	–	–	–	–
	COVID‐19 cases: smoothed	0.216	–	–	–	–
	COVID‐19 cases: smoothed and lag	–	–	–	–	–
	Number of foreign tourists linear	0.005	−0.171	−0.289	−0.054	1171.824
	Number of foreign tourists: smoothed	–	–	–	–	–

Abbreviations: AIC, akaike information criterion; CI, confidence interval; GRSI, Government Response Stringency Index; HM, hypermarkets; SM, supermarkets.

^a^ *p*‐value of COVID‐19 related term in the model.

^b^ Rate of change for linear models is a parameter estimate; for models with smooth we computed average rate of change as an illustration because it reduces the complexity and variability of the functional relationship to a single metric.

^c^ Confidence intervals are provided for linear terms, for smoothed terms we provide graphical representation in Appendix (Figures A7, A8, A9, A10, A11, A12, A13).

The number of COVID‐19 cases was not a significant predictor in any of the presented models. The models using total sales revenues show that the total amount of alcohol sold decreased with the increase of the GRSI score and tightening of alcohol outlet restrictions. The decrease in the number of overnight tourists was associated with a decrease in overall alcohol sales. The lowest AIC total sales revenues model is the GRSI model with smooth.

Regarding retail sales, we did not identify any model with COVID‐19 cases as a significant predictor. The retail sales data show that the overall amount of alcohol sold in retail outlets increased with the increase of the GRSI and tightening of the outlet restrictions. Models that include GRSI also show the lowest AIC.

## 4 DISCUSSION

The total alcohol sales revenue data show that the overall amount sold in Czechia decreased 13% with the imposition of the COVID‐19 measures. Like other studies [6, 18, 39], our study shows that the changes in alcohol sales varied directly depending on the COVID‐19‐related restrictions. Contrary to studies from North America [6, 18], we did not find any relationship between alcohol sales and the number of new COVID‐19 cases.

The models that include GRSI had lower AIC than the models with intervention variables and thus had higher quality, suggesting that general restrictions limiting movement and gathering (measured by GRSI) were having more of an influence on alcohol sales than only alcohol outlet‐specific restrictions (measured by intervention variables). This is consistent with a Norwegian survey [21], which found that respondents attributed their decreased drinking to fewer social occasions, and with the observation that social restrictions might be an important driver behind the overall decrease in consumption [2]. Furthermore, the decline in alcohol consumption during COVID‐19 was observed in heavy episodic drinking [2]. This observation may also apply to Czechia, where alcohol consumption plays an important role in Czech culture and in social encounters. The change in consumer behaviour related to different types of COVID 19 restrictions was also observed [39]. Relating this results to the alcohol availability theory [28], it highlights that in Czechia the social aspect of alcohol availability is important one.

We found that models including GRSI had lower AIC than models including intervention variables, suggesting that the former models are better in representing the data than models including variables usually used in time series intervention analyses (intervention variables). Thus, the GRSI should be considered a suitable variable in future analyses. Measures of different COVID‐19 restrictions should also be considered.

We focused on the effect of the number of overnight stays of foreign tourists, which was found to be a significant predictor. However, the models that included other predictors proved to be of a higher quality. Before the COVID‐19 pandemic, a study of the role of alcohol consumption by tourists in Barcelona, Spain concluded that there was a strong association between tourism pressure and alcohol exposure, that is, the more tourism is promoted through development of the late‐night economy, the more alcohol is available to the local population at restaurants and bars, thus adding to per capita consumption beyond what is purchased by foreign tourists [40]. Thus, any increases in alcohol consumption due to tourism are likely to increase the level of alcohol problems in the areas surrounding pubs, bars and clubs [41, 42]. This may also apply to the spread of communicable diseases like COVID‐19.

The results of the retail sales analyses show that the amount of alcohol sold increased in relation to the COVID‐19 related measures in retail shops. Thus, the overall decrease observed in the total sales revenues was probably the result of the decrease in alcohol sold in restaurants, which was partially supplemented by the increase of sales in all segments of the retail sector. Many people may have changed their drinking habits to buying more alcohol for home consumption with family and friends (although there were curfews, visits to other people's homes were not effectively controlled). Our results are in line with a study [39] that also found a decrease in visits to drinking places (restaurants, bars) in several states in the United States due to the lockdown restrictions.

One strength of our study is that we tested models for multiple COVID‐19‐related variables. To our knowledge, this is the only study that uses the GRSI and the number of foreign overnight tourists to evaluate COVID‐19 measures in relation to alcohol sales. Another strength of our study is that it uses data from two independent data sources capturing alcohol sales, and we had two indicators of COVID‐19 measures, one focusing on the alcohol outlets and the other on limiting social mobility and gatherings. However, each of these sources has its limits as described in the Methods section. The major limitation of our analysis is that we cannot separate the effects of some variables and events because they overlapped, and the variables are highly correlated. Furthermore, including more variables into one complex model could result in unreliable models using the autocorrelation specifications and smoothed variables. We addressed this issue by comparing different models. Moreover, the analysis is at the ecological, not the individual level. The estimates provided in the case of models using the variables representing outlet shop restrictions might be considered less reliable because the length of the time series before the interventions (pre‐epidemic measures) was rather short. Unlike some of the other studies [39], we did not examine in detail types of alcohol sold or the geographical differences between different regions of Czechia. Because this study's interrupted time series analyses do not include a control series, causal inferences that the interventions were the only or the main cause of the changes in alcohol sales requires an additional assumption that there are no other confounds that occurred close in time with the interventions.

## 5 CONCLUSION

The findings indicate that 13% decline in total alcohol sales resulting from the COVID‐19 restrictions was not just the result of reduced tourism and/or restrictions on physical availability of alcohol. General restrictions on social encounters also contributed. This conclusion is consistent with central role that beer and other drinks play in Czech social encounters [43, 44, 45]. Thus, the decrease in alcohol sales during the COVID‐19 restrictions might have been mediated by the reduced availability of social occasions where drinking would have occurred [21]. Comparing the impact of COVID‐19 restrictions in different countries, we should consider the role that alcohol plays in the particular drinking culture as an agent of socialisation. This is particularly important when planning public health interventions. The above factors should be taken into account in future research comparing trends in different countries. Finally, this study demonstrates the value not only of time series data, but also of using different measures of alcohol sales and summary measures of public health restrictions, while taking relevant subpopulations (e.g., tourists) into account.

## AUTHOR CONTRIBUTIONS

Each author certifies that their contribution to this work meets the standards of the International Committee of Medical Journal Editors.

## CONFLICT OF INTEREST STATEMENT

Benjamin Petruželka has no interests to declare. Miroslav Barták has no interests to declare. Vladimir Rogalewicz has no interests to declare. Thomas F. Babor has no interests to declare.

## Supporting information


**Data S1.** Supporting information.

## Figures and Tables

**FIGURE A7:**
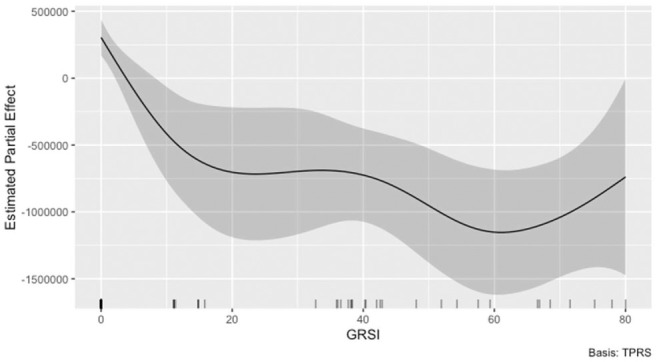
Partial effect of GRSI on alcohol sales (95% CI).

**FIGURE A8:**
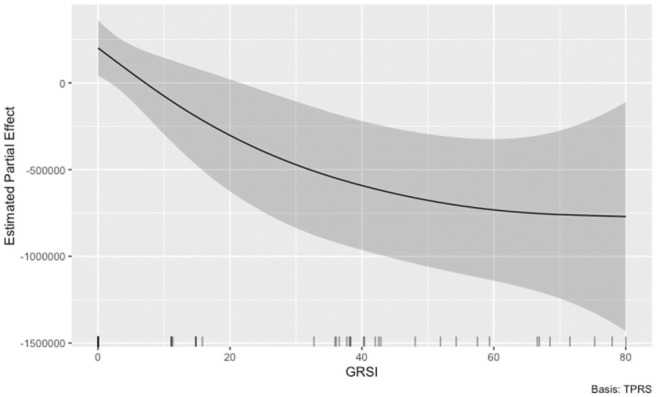
Partial effect of GRSI with lag on overall sales (95% CI).

**FIGURE A9:**
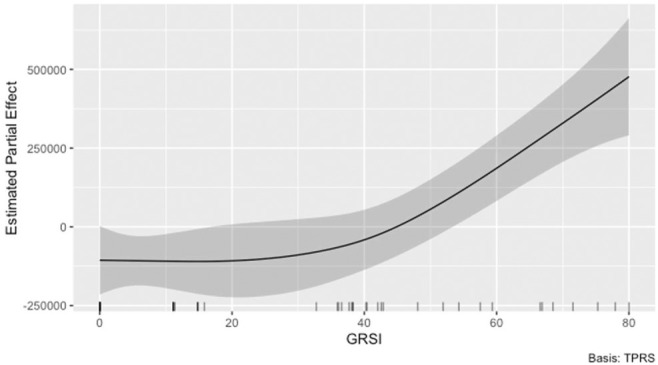
Partial effect of GRSI on total retail sales (95% CI).

**FIGURE A10:**
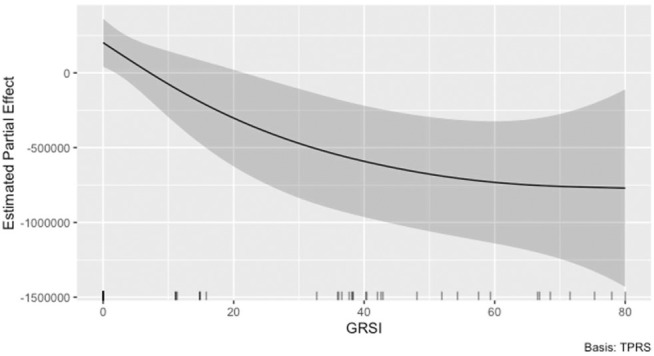
Partial effect of number of tourists on total retail sales (95% CI)

**FIGURE A11:**
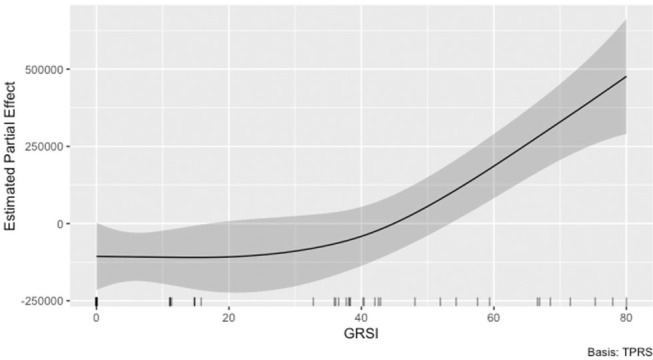
Partial effect of GRSI on hh/sm sales (95% CI).

**FIGURE A12:**
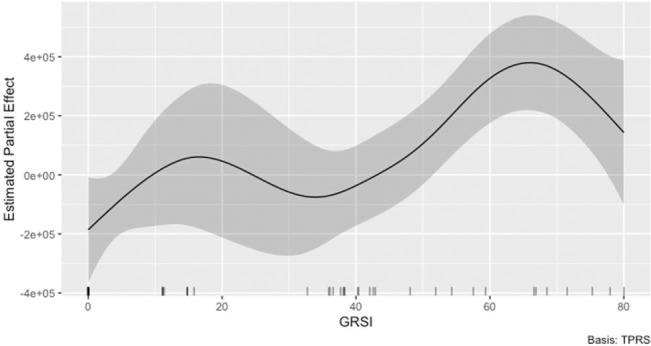
Partial effect of GRSI with lag on hh/sm sales (95% CI).

**FIGURE A13:**
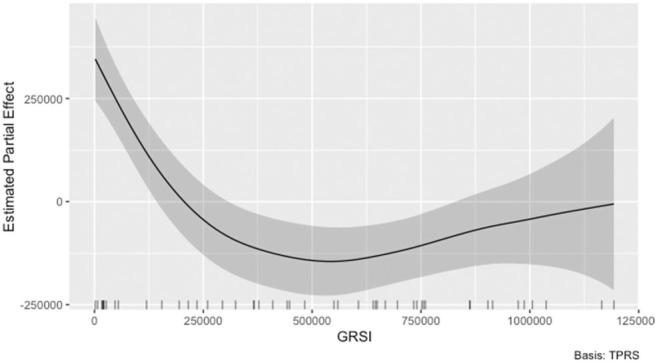
Partial effect of number of tourists on total retail sales (95% CI)

## Appendix

**TABLE A1:** Governmental restrictive measures affecting alcohol sales in the Czech Republic related to the COVID‐19 pandemic.

Lockdowns resulting from the first wave of the pandemic (March to May 2020)	
Restrictions on mobility and assembly	The state of emergency was in effect from 12 March to 17 May. The free movement of people was restricted to necessary cases. All sport and cultural events were cancelled. All types of schools were closed from 11 March until virtually the end of June (the beginning of the holidays), although from 11 May certain activities were allowed on a limited basis.
On‐premise alcohol outlets (restaurants, bars, cafes, etc.)	All hospitality facilities were closed from 14 March until 25 May 2020; although takeaway sales through windows were possible, they were not allowed to sell alcoholic beverages.
Tourism	The borders were closed on 16 March 2020. Travelling was slightly relaxed on 24 April 2020 but cross‐border tourism was still significantly reduced.
Intermediate period (June to September 2020)	
Tourism	Tourism was significantly reduced.
Lockdowns resulting from the second wave of the pandemic (October 2020 to April 2021)	
Restrictions on mobility and assembly	A second state of emergency was declared with similar restrictions from 5 October 2020, and lasted until 11 April 2021. Moreover, all companies were recommended to allow work from home whenever possible during this period. All schools were closed from 12 October mostly until 16 May 2021 in elementary schools and until school holidays (end of June) in secondary and higher education (there were some partial exceptions valid for particular types of education/periods/districts during this school lockdown).
Curfews	On 11 February 2021, a curfew was declared in three districts. The curfew was extended to the entire territory of the Czech Republic on 26 February 2021 and lasted until 12 April 2021.
On‐premise alcohol outlets (restaurants, bars, cafes, etc.)	These facilities were closed on 13 October 2020 (with the exception for takeaway sales). Only on 17 May 2021, the restaurants were allowed to open (beer) gardens with at most four persons at a table, while the inside rooms of restaurants were open on 31 May (a proof of vaccination required).
Drinking alcohol in public places	A ban of drinking alcohol in public places was in force and strictly enforced by the police from 13 October 2020 to 12 April 2021.
Tourism	With vaccination starting in December 2020, similarly like in other (European) countries, the travel restrictions had been stepwise waived for persons with vaccination proof.
Intermediate period (May to November 2021)	
Everyday life, social activities	The majority of mobility restrictions were lifted in the spring and summer 2021.
Tourism	The travel restrictions had been stepwise waived for persons with vaccination proof, so that holidays 2021 witnessed a cautious restart of cross‐border tourism.
Lockdowns resulting from the third wave of the pandemic (November 2021 to December 2021)	
Restrictions on mobility and assembly, on‐premise alcohol outlets	The third state of emergency was declared on 25 November and was effective until 25 December 2021 and again from 29 December 2021 to 2 January 2022. During this time, the provision of services has been restricted but not prohibited. The only exception were Christmas markets, which were banned.
Drinking alcohol in public places	A ban of drinking alcohol in public places was in force and strictly enforced from 25 November 2021 to 25 December 2021.
Tourism	No restriction for vaccinated.
Final period (January to April 2022)	
Restrictions on mobility and assembly, everyday life, social activities, on‐premise alcohol outlets, cross‐border travel and tourism	On 17 December 2021, a new Government decided on a softening of the COVID‐19 measures from 3 January 2022 and a timetable for their phasing out.

*Note*: The government finally lifted the pandemic alert on 3 May 2022, which meant the termination of all full‐scale measures in the Czech Republic.

**TABLE A6:** Correlation between COVID‐19 and tourism related variables.

	Stringency index	Stringency index: Lag	COVID‐19 cases	COVID‐19 cases: Lag	Restrictions on outlets	Restrictions on outlets: Lag	Number of tourists
Stringency index		0.9718046	0.9691171	0.9441811	0.7120985	0.6912692	−0.6552792
Stringency index: lag	0.9718046		0.9582526	0.9704556	0.6616435	0.7195209	−0.6345403
COVID‐19 cases	0.9691171	0.9582526		0.9835830	0.7103354	0.6808285	−0.6009749
COVID‐19 cases: lag	0.9441811	0.9704556	0.9835830		0.6809207	0.7203817	−0.5833614
Restrictions on outlets	0.7120985	0.6616435	0.7103354	0.6809207		0.7944032	−0.5460499
Restrictions on outlets: lag	0.6912692	0.7195209	0.6808285	0.7203817	0.7944032		−0.5156946
Number of tourists	−0.6552792	−0.6345403	−0.6009749	−0.5833614	−0.5460499	−0.5156946	

### A14. Optimal models and modelling strategy

Regarding total sales revenues, the baseline model includes trend variable and smoothed seasonal variable. For the seasonal variable in the baseline model the smooth basis type was first specified as cyclic cubic regression splines (cc) and k = 12. However, the k.check() *p*‐value was <0.001. Switching the basis to thin plate regression splines (tp) keeping k = 12 provided better results *p* = 0.145. After that, the following autocorrelation structure was specified for baseline model (*p* = 2, q = 3). The check of the splines revealed, in models using COVID‐19 cases and the number of tourists, that use of the smooths does not provide suitable models (i.e., the relationship is linear) and, thus, these models were not further used and Table 3 does not include information about these models (see R syntax appendix for model splines and k‐check values). For other smoothed variables, we used thin plate regression splines (tp) and k.check() showed reasonable results and therefore we used the k values estimated by the software.

Regarding total retail sales, the baseline model includes trend variable and smoothed seasonal variable. For the seasonal variable in the baseline model the smooth basis type was first specified as cyclic cubic regression splines (cc) and k = 12. However, the k.check() *p*‐value was <0.001. Switching the basis to thin plate regression splines (tp) keeping k = 12 provided better results *p* = 0.582. After that, the following autocorrelation structure was specified for baseline model (*p* = 1, q = 1). The check of the splines and the k‐check revealed for model using GRSI with lag and models using COVID‐19 cases that using the smooths is not suitable and, thus, these models were also not further used (see R syntax appendix for model splines and k‐check values). For other smoothed variables, we used thin plate regression splines (tp) and k.check() showed reasonable results and therefore we used the k values estimated by the software.

Regarding HM/SM retail sales, the baseline model includes trend variable and smoothed seasonal variable. For the seasonal variable in the baseline model the smooth basis type was first specified as cyclic cubic regression splines (cc) and k = 12. However, the k.check() *p*‐value was <0.001. Switching the basis to thin plate regression splines (tp) keeping k = 12 provided better results *p* = 0.66. After that, the following autocorrelation structure was specified for baseline and thus for all models (*p* = 1, q = 0). The k‐check revealed for model including COVID‐19 cases and lag that using the smooth is not suitable and, thus, these models were also not used further (see R syntax appendix for model splines and k‐check values). For other smoothed variables, we used thin plate regression splines (tp) and k.check() showed reasonable results and therefore we used the k values estimated by the software. The exception to this was the smoothed number of tourists and we set the k = 20 which led to reasonable results.

Regarding small shops retail sales, the baseline model includes smoothed trend variable and smoothed seasonal variable. For the seasonal variable in the baseline model the smooth basis type was first specified as cyclic cubic regression splines (cc) and k = 12. However, the k.check() *p*‐value was <0.001. Switching the basis to thin plate regression splines (tp) keeping k = 12 provided better results *p* = 0.078. The smoothed trend variable with basis specified as thin plate regression splines (tp) showed reasonable results (*p* = 0.802). After that, we did not identify any autocorrelation structure. The check of the splines and k‐check revealed for all models including smooths except model including COVID‐19 cases that using the smooths is not suitable and, thus, these models were also not further used (see R syntax appendix for model splines and k‐check values). For other smoothed variables, we used thin plate regression splines (tp) and k.check() showed reasonable results and therefore we used the k values estimated by the software (except smoothed variable of number of COVID‐19, k value was set as 20).
